# Bilateral Subareolar Epidermal Inclusion Cysts of the Breast After Prior Surgery for Mastitis: Differing Sonographic Appearances of Ruptured and Unruptured Cysts

**DOI:** 10.3390/diagnostics16213577

**Published:** 2026-10-29

**Authors:** Kyung Jin Nam, Ha Young Park, Gi Won Shin

**Affiliations:** 1 Department of Radiology, Pusan National University School of Medicine, Pusan National University Yangsan Hospital, Yangsan 50612, Republic of Korea; kj-violet@hanmail.net; 2 Department of Pathology, Busan Paik Hospital, Inje University College of Medicine, Busan 47392, Republic of Korea; 3 Department of Radiology, Busan Paik Hospital, Inje University College of Medicine, Busan 47392, Republic of Korea

**Keywords:** epidermal inclusion cyst, breast, ultrasonography, onion-ring sign, shear-wave elastography, nipple discharge

## Abstract

**Background and Clinical Significance**: An epidermal inclusion cyst (EIC) of the breast is an uncommon benign lesion, and bilateral subareolar involvement is exceptionally rare. Because a ruptured EIC can mimic malignancy or a primary infection, recognition of its imaging spectrum is clinically important. **Case Presentation**: A woman in her 40s presented with bilateral palpable subareolar masses and bloody, purulent nipple discharge, which had developed gradually over several months, ten years after bilateral surgery for mastitis. Mammography showed a right subareolar focal asymmetry with nipple–areolar thickening and a left circumscribed hyperdense mass. Sonography demonstrated a right mass with a preserved lamellated “onion-ring” pattern that proved unruptured at excision, and a left heterogeneously hypoechoic mass with small cystic changes that proved ruptured, with microabscess and hemorrhage; both masses were avascular on color Doppler, and the left mass showed focally high stiffness (peak 214.7 kPa) on shear-wave elastography. Surgical excision confirmed bilateral EIC. **Conclusions**: The coexistence of ruptured and unruptured cysts in one patient illustrates their differing sonographic appearances. Recognizing the onion-ring sign and integrating multiparametric ultrasound help distinguish benign EICs from malignancy or infection, while underscoring the need for histologic confirmation.

## 1. Introduction

Epidermal inclusion cysts (EICs) are benign, keratin-filled lesions lined by stratified squamous epithelium that most often arise in the skin of the scalp, face, neck, and trunk [1]. They are uncommon in the breast, where they usually involve the cutaneous or subcutaneous layer; a deep parenchymal or subareolar location is unusual [2,3,4]. Breast EICs may be congenital or acquired after trauma, prior surgery, reduction mammoplasty, augmentation, or needle procedures, and squamous metaplasia of ductal epithelium has also been proposed as a mechanism [1,2,5]. Although benign, EICs may rupture and incite an inflammatory or suppurative reaction, and rare malignant transformation into squamous cell carcinoma has been described; consequently, they can mimic malignancy both clinically and on imaging [6,7]. Bilateral subareolar EICs are exceedingly rare [5]. We report a case of bilateral subareolar EICs arising years after bilateral breast surgery, in which the intact and ruptured cysts showed contrasting sonographic appearances, and we review the current imaging literature on this entity.

## 2. Case Presentation

A woman in her 40s presented with bilateral palpable subareolar masses and bilateral bloody, purulent nipple discharge. The masses had gradually enlarged over several months and were larger on the left, and the bilateral nipple discharge had developed progressively and in parallel; there was no associated pain, erythema, or fever. Ten years earlier she had undergone bilateral breast surgery for mastitis; by the patient’s recollection this was presumed to be incision and drainage, although the operative and pathology records were no longer available. On review of the available medical records, the patient was parous (two children) and a former smoker; however, the timing of her deliveries relative to the mastitis episode and her breastfeeding history were not documented, so an overlap with lactation could not be entirely excluded. The exact type of the prior mastitis therefore could not be definitively established; nonetheless, in the absence of any documented association with pregnancy or lactation, and given her smoking history—which is characteristically associated with periductal mastitis—the features favored a non-puerperal (periductal) etiology. There was no personal or family history of breast malignancy.

**Figure 1:**
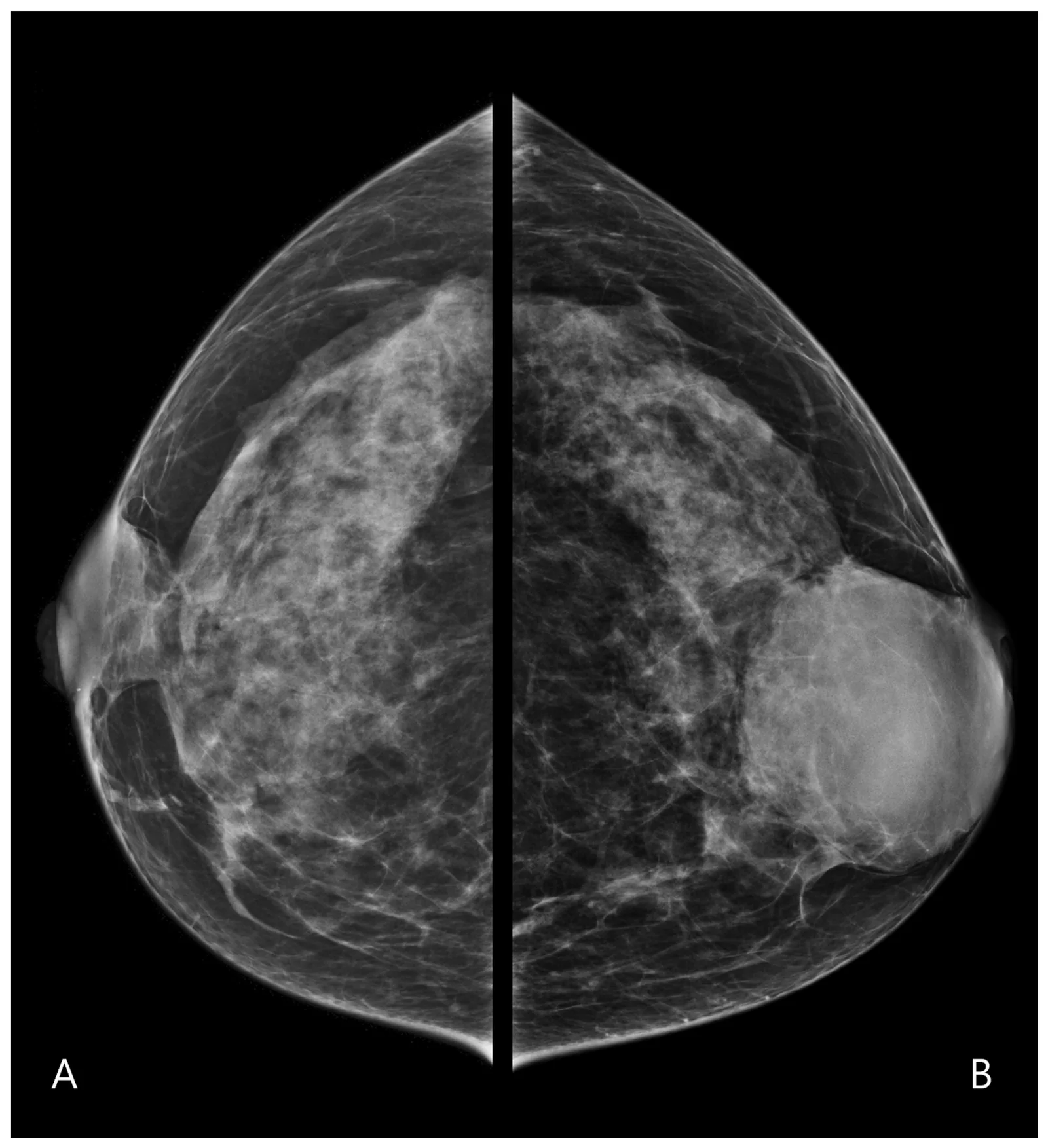
Bilateral mammography (Selenia Dimensions, Hologic). (**A**) The right subareolar region shows a focal asymmetry with thickening of the nipple–areolar complex. (**B**) The left subareolar region shows a circumscribed, round, hyperdense mass.

**Mammography** (Selenia Dimensions, Hologic, Marlborough, MA, USA) demonstrated a focal asymmetry in the right subareolar region with thickening of the nipple–areolar complex, and a circumscribed, round, hyperdense mass in the left subareolar region (Figure 1).

**Figure 2:**
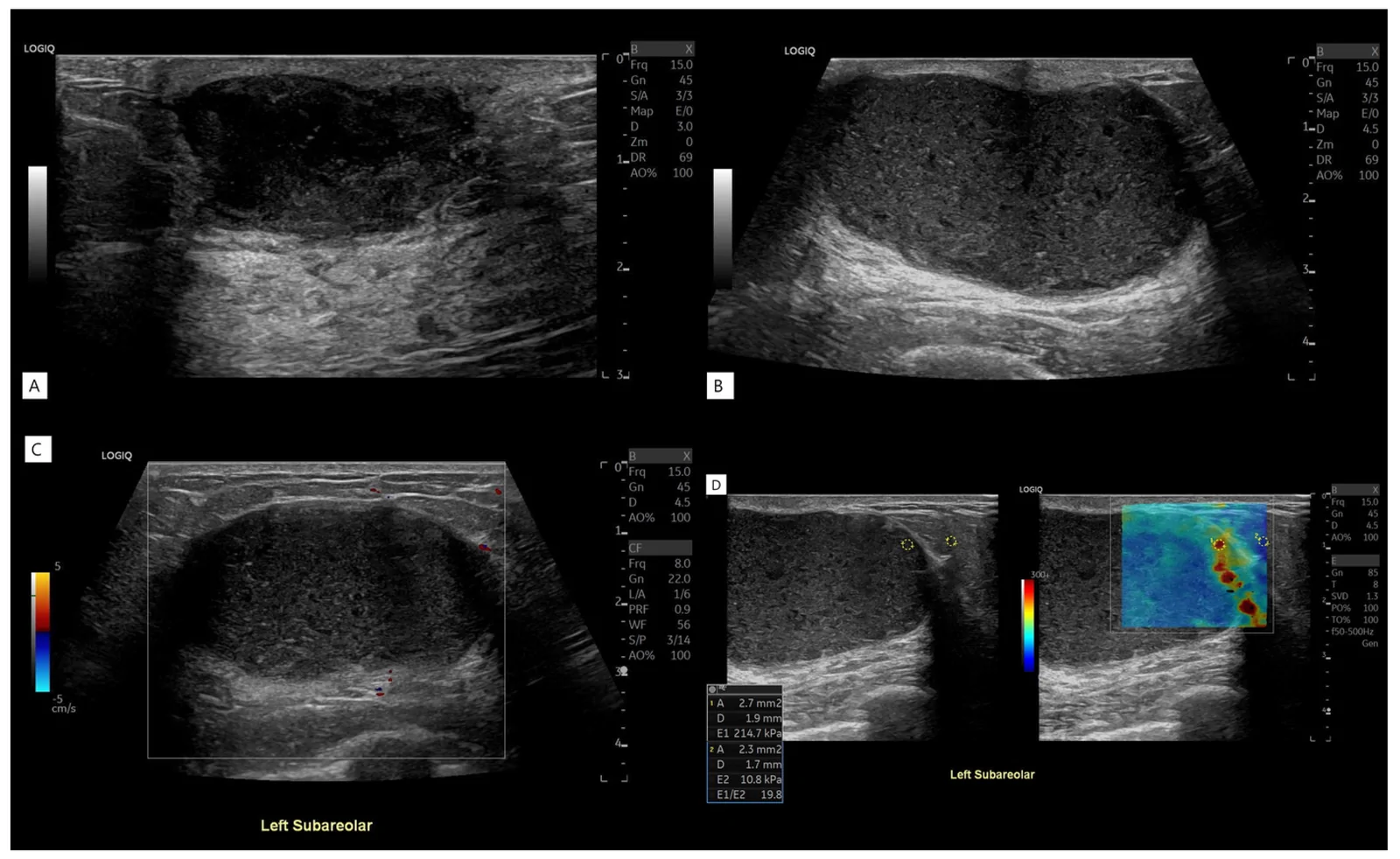
Initial ultrasonography (LOGIQ E10, ML4–20 MHz linear transducer, GE Healthcare). (**A**) Right subareolar 2.7–cm lobulated, partially indistinct, mixed solid–and–cystic mass with posterior acoustic enhancement and a concentric onion–ring pattern. (**B**) Left subareolar 5.7–cm circumscribed, oval, heterogeneously hypoechoic mass with multiple small internal cystic spaces. (**C**) Color Doppler sonography of the left mass shows no internal vascularity. (**D**) Shear–wave elastography of the left mass shows focally high stiffness; a region of interest over the solid portion measured 214.7 kPa and a reference region over adjacent subcutaneous fat measured 10.8 kPa (lesion–to–fat ratio, 19.8).

**Sonography** (LOGIQ E10, ML4-20 MHz linear transducer, GE Healthcare, Chicago, IL, USA) showed, in the right subareolar region, a 2.7-cm lobulated, partially indistinct, mixed solid-and-cystic mass with posterior acoustic enhancement and a concentric “onion-ring” appearance reflecting lamellated keratin (Figure 2A). In the left subareolar region there was a 5.7-cm circumscribed, oval, heterogeneously hypoechoic mass containing multiple small internal cystic spaces (Figure 2B). On color Doppler sonography, neither the right nor the left subareolar mass showed internal vascularity (the left-sided image is shown in Figure 2C). On shear-wave elastography, the left subareolar mass demonstrated focally high stiffness; one region of interest was placed over the solid portion of the mass showing the highest stiffness, which measured 214.7 kPa, and a second region of interest was placed over the adjacent subcutaneous fat as a reference, which measured 10.8 kPa, giving a lesion-to-fat stiffness ratio of 19.8 (Figure 2D). Shear-wave elastography was not performed on the right lesion. High stiffness is not specific and may also be seen in malignant or inflammatory lesions (see Discussion). The nipple discharge was present bilaterally and was not more prominent on the ruptured (left) side; discharge cytology and galactography were not performed. Although the progressively enlarging masses, the bloody nipple discharge, the partially indistinct right-sided margin, and the heterogeneous internal echotexture raised the possibility of a more suspicious assessment and prompted consideration of immediate tissue diagnosis, the known history of prior bilateral breast surgery for mastitis and the overall imaging appearance favored a benign or inflammatory process; both lesions were therefore assessed as BI-RADS category 3 (probably benign), for which short-interval follow-up was initially recommended.

**Figure 3:**
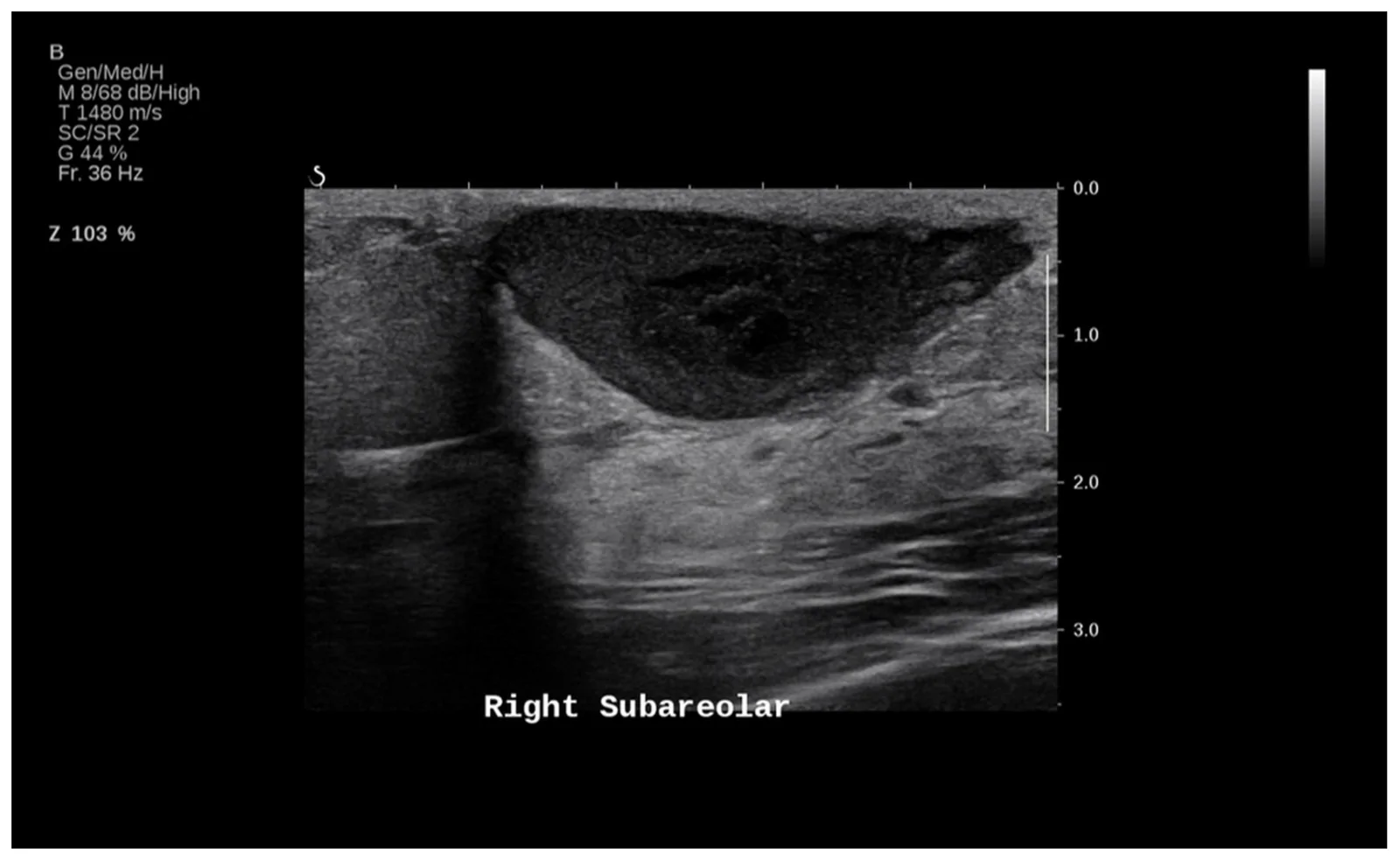
One-month follow-up ultrasonography of the right breast (Aixplorer, SuperSonic Imagine, Aix-en-Provence, France). Compared with the initial study, the subareolar mass shows a more clearly defined concentric (onion-ring) pattern, consistent with a keratin-containing epidermal inclusion cyst.

At one-month follow-up sonography (Aixplorer, SuperSonic Imagine, Aix-en-Provence, France), the symptoms had not improved and the right mass appeared slightly larger; the right subareolar mass also showed a more clearly defined concentric ring pattern (Figure 3).

**Figure 4:**
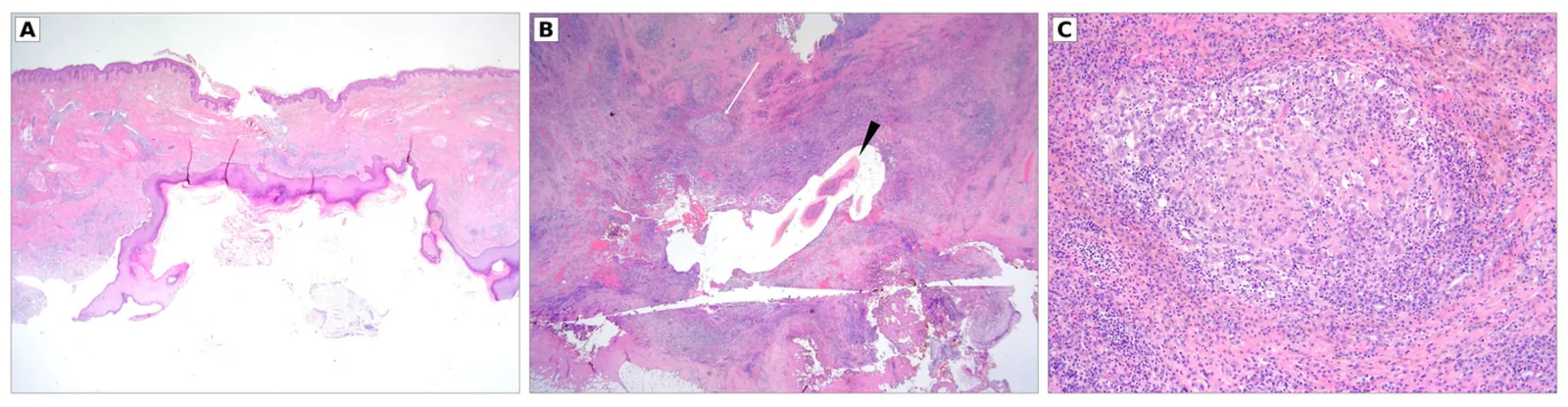
Histopathologic photomicrographs (hematoxylin–eosin). (**A**) The right (unruptured) lesion (×12.5) shows a cyst lined by keratinizing stratified squamous epithelium with a well-preserved lining and laminated keratin. (**B**) The left (ruptured) lesion (×12.5) shows disruption of the cyst wall with detached keratinizing squamous lining cells and lamellated keratin within the residual cyst lumen (black arrowhead); keratin that has extravasated into the surrounding tissue through the disrupted wall has elicited a foreign-body reaction (white arrow). (**C**) Higher-power view of the white-arrow region in (**B**) (×100): foreign-body granuloma formed in reaction to the extravasated keratin in the surrounding tissue.

Because the masses were bilateral, palpable, progressively enlarging, and accompanied by persistent bloody, purulent nipple discharge that did not improve on short-interval follow-up—with the right mass appearing slightly larger—surgical excision of both lesions was performed for definitive diagnosis and symptom relief. Core-needle biopsy was not performed first, as the lesions were considered probably benign on imaging and biopsy of a suspected EIC carries a risk of provoking rupture and inflammation. Histopathology confirmed an epidermal inclusion cyst on the right, and a ruptured epidermal inclusion cyst with microabscess, hemorrhage, and granulation tissue on the left (Figure 4). The findings of rupture and inflammation were confined to the left cyst, and no definite concurrent ductal lesion was identified on the excision specimens. After excision, the patient’s symptoms improved and the bilateral nipple discharge resolved. Over two years of postoperative ultrasound follow-up, there was no recurrence and no postoperative complications.

## 3. Discussion

### 3.1. Epidemiology and Pathogenesis

**Figure 5:**
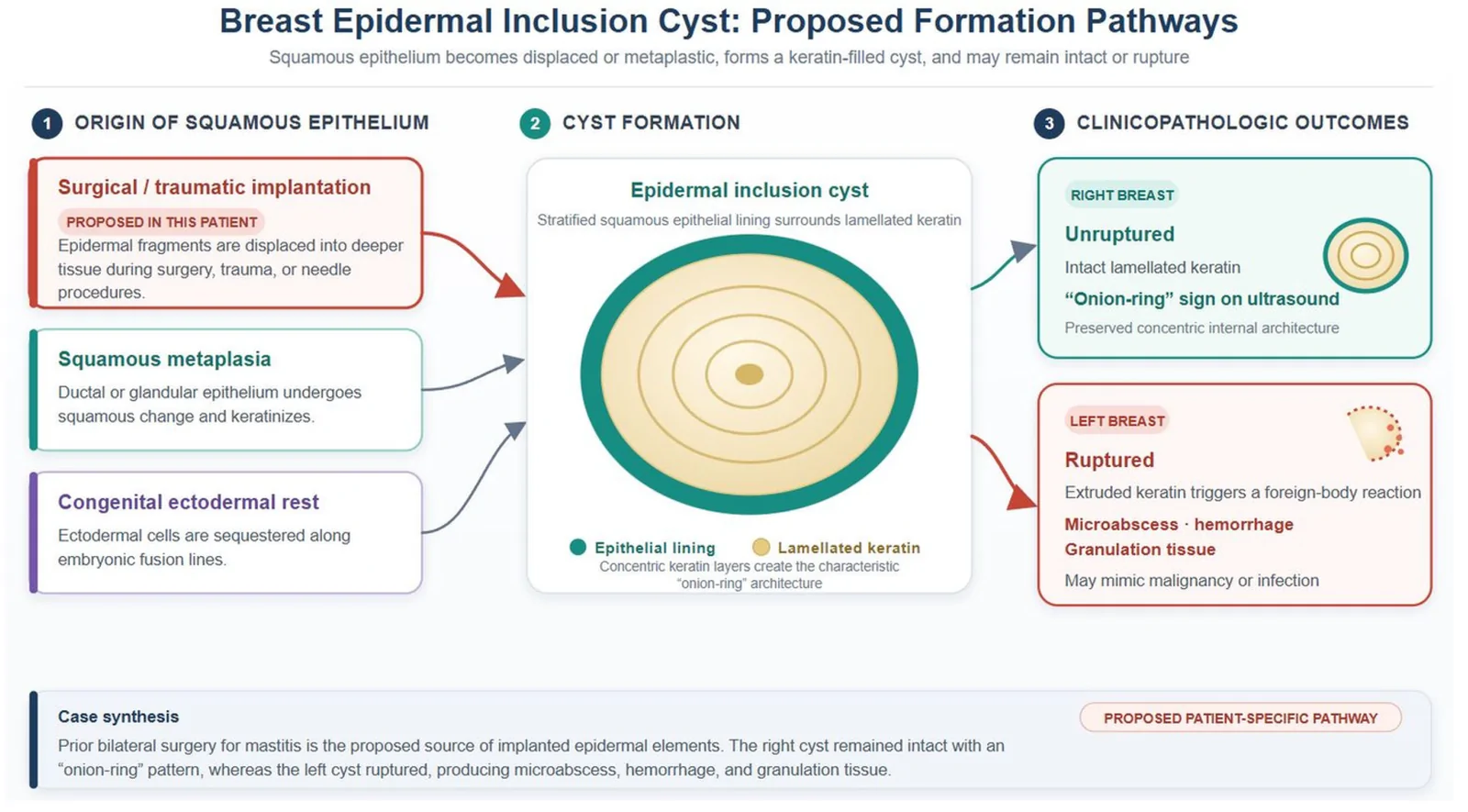
Schematic of the proposed mechanisms of breast epidermal inclusion cyst formation. Displaced or metaplastic squamous epithelium forms a keratin-filled cyst that may remain intact—retaining the lamellated “onion-ring” architecture—or rupture, extruding keratin and provoking a foreign-body reaction with microabscess, hemorrhage, and granulation tissue.

EICs are among the most common cutaneous cysts but are rare in the breast, where they typically involve the skin or subcutaneous tissue; a subareolar or deep parenchymal location is distinctly unusual and accounts for much of the diagnostic difficulty in this region [3]. Bilateral subareolar disease, as in the present case, is rarely encountered [5,8]. Breast EICs may be congenital, arising from ectodermal sequestration, or acquired; implantation of epidermal elements during trauma or surgery—including reduction mammoplasty—is a well-recognized mechanism, and squamous metaplasia of ductal epithelium has also been proposed [2,5]. A recent series correlating ultrasound and CT with surgical findings similarly emphasized traumatic or surgical implantation and the tendency of these cysts to become infected or to rupture as they enlarge [1]. Although surgery for mastitis (the type was not definitively established but was favored to be non-puerperal/periductal in this patient)—such as incision and drainage—is typically directed at a parenchymal or subareolar inflammatory collection rather than the epidermis, any procedure that creates a cutaneous or subareolar incision can displace fragments of surface epithelium into the deeper tissue and provide a route for implantation. In this patient, the prior surgery was, by her recollection, presumed to be incision and drainage, but the operative and pathology records were no longer available; the surgically related, bilateral distribution therefore remains a plausible but unconfirmed explanation (Figure 5).

### 3.2. Imaging Features

On sonography, the most useful clue to an EIC is the onion-ring, or concentric ring, pattern, which corresponds to lamellated keratin within the cyst and was first emphasized as a specific sign by Crystal and Shaco-Levy [9,10]. Sonographic–pathologic correlation studies have shown that the internal echoes reflect variable arrangements of keratin, ranging from homogeneously hypoechoic to laminated or nodular patterns [11]. Feature-based diagnostic models have since been proposed to distinguish EICs from other superficial soft-tissue lesions, including the “submarine sign” of a deep protruding portion [12]. When an EIC ruptures, this organized architecture is lost: ruptured cysts tend to become lobulated or ill-defined, lose the halo, show a more heterogeneous echotexture, and may develop pericystic changes and peripheral or intermediate Doppler vascularity owing to surrounding inflammation and granulation tissue [13,14]. In a comparative sonographic series, Yuan et al. reported the breast as one of the more common sites of rupture and confirmed these grayscale and Doppler differences [14]. On MRI, EICs show variable signal with restricted diffusion related to keratin, and rupture is associated with peri-lesional inflammatory change [15]. In the present case, the intact right cyst retained the onion-ring architecture and was avascular, whereas the ruptured left cyst lost the pattern and showed inflammatory features. For the left lesion, the clinical, radiologic, and pathologic findings were concordant: the heterogeneously hypoechoic appearance with small cystic spaces and loss of the organized onion-ring architecture corresponded to disruption of the cyst wall, with extruded keratin, microabscess, hemorrhage, and a foreign-body granulomatous reaction on histopathology. The bilateral bloody, purulent nipple discharge is most plausibly attributable to the subareolar cysts and the associated inflammation—particularly the ruptured, suppurative left lesion—given the subareolar location adjacent to the ductal system; however, no definite concurrent ductal pathology was identified on histopathology and discharge cytology and galactography was not performed; a coexisting ductal process cannot be entirely excluded. Notably, the intact right cyst was itself lobulated and partially indistinct—features that overlap with the ruptured profile—underscoring that shape and margin alone are imperfect discriminators and that the keratin-related onion-ring sign was the more dependable indicator of benignity. The differences are summarized in Table 1.

### 3.3. Color Doppler and Elastography

The absence of detectable internal vascularity in both lesions on color Doppler was compatible with the cystic nature of the masses but is not specific for benignity, since some malignant lesions may show little or no detectable flow [14]. Shear-wave elastography was performed on the left (ruptured) mass only and showed focally high stiffness, with a peak value of 214.7 kPa. Shear-wave elastography quantifies tissue stiffness (reported here in kilopascals); higher values indicate stiffer tissue, and although no universal threshold exists for superficial lesions, values of this magnitude overlap with the range reported for suspicious or malignant breast lesions [16,17]. The high stiffness of the ruptured lesion likely reflects its mixed solid composition—compact keratin together with microabscess, hemorrhage, and surrounding foreign-body granulomatous inflammation—rather than keratin alone. Increased stiffness is nonetheless well recognized in epidermal cysts, reflecting compact lamellated keratin, and epidermoid lesions can be as stiff as—or stiffer than—some malignant tumors, representing a potential pitfall [16,17]; elastographic measurements in these cysts are also reproducible across strain and shear-wave techniques [18]. Elastography has been reported to help differentiate ruptured from unruptured cysts [19] and to distinguish a ruptured cyst from a superficial abscess [20] in larger series; however, because elastography was applied to only the left lesion in our case, no conclusion about its discriminatory value can be drawn here. Overall, because such hard elastographic values overlap with those of malignancy and infection, stiffness should be interpreted together with the grayscale onion-ring sign rather than in isolation, reinforcing the need for histologic confirmation.

**Table 1:** Comparison of imaging features of unruptured and ruptured breast epidermal inclusion cysts synthesized from the literature [13,14,15,21], with the findings in the present case. EIC, epidermal inclusion cyst.

Feature	Unruptured EIC	Ruptured EIC	Present Case (Right/Left)
**Shape**	Oval or round	Lobulated or irregular	Right lobulated/left oval
**Margin**	Well circumscribed	Ill- or poorly defined	Right partially indistinct/left circumscribed
**Halo**	Often present	Often absent	Not conspicuous
**Echotexture**	Homogeneous, hypoechoic	Heterogeneous	Right mixed solid–cystic/left heterogeneous with small cysts
**Onion-ring (lamellated keratin) sign**	Frequently present	Usually lost	Right present (clearer at follow-up)/left absent
**Posterior acoustic enhancement**	Common	Common	Right present
**Color Doppler**	Avascular	Peripheral/intermediate flow possible	Both lesions: no internal vascularity
**Elastography (stiffness)**	Stiff—compact keratin	Variably stiff—mixed keratin, microabscess, hemorrhage, and granulomatous inflammation	Left: peak 214.7 kPa (SWE; right not performed)
**Surrounding tissue**	None	Inflammation, foreign-body reaction, microabscess	Left: microabscess, hemorrhage, granulation tissue

### 3.4. Differential Diagnosis and Management

Ruptured subareolar EICs are particularly prone to be mistaken for malignancy or for primary infectious processes, as described by Whang et al. and, more recently, in other subareolar cases [21,22]. The differential diagnosis of a subareolar mass with indistinct margins and bloody or purulent discharge includes subareolar abscess, primary breast carcinoma, and benign masses such as fibroadenoma [23].

Periductal mastitis and its recurrent fistulizing form, Zuska disease, deserve particular consideration here, because they classically present with subareolar masses, nipple discharge (which may be bloody or purulent), and a tendency to recur—features that overlap closely with a ruptured subareolar EIC. Some features may help distinguish them: Zuska disease is strongly associated with cigarette smoking and typically shows dilated subareolar ducts, periductal inflammation, and recurrent fistula formation to the areolar skin [24], whereas a ruptured EIC tends to show a discrete keratin-containing mass—often with residual lamellated “onion-ring” architecture in an unruptured portion or, as here, in the contralateral lesion—and a foreign-body granulomatous reaction on histology. In practice, however, the two may be indistinguishable on imaging alone, and histopathology is often required for a definitive distinction, as in our patient.

When sonography shows the classic onion-ring sign, a benign EIC can be strongly suggested; however, as this case illustrates, ruptured or atypical lesions may lose this characteristic pattern and mimic malignancy or infection, so imaging alone may be insufficient for a definitive diagnosis in symptomatic or atypical subareolar lesions. It should be emphasized that a BI-RADS category 3 (probably benign) assessment is not generally appropriate for an enlarging mass accompanied by suspicious clinical symptoms such as bloody nipple discharge; in the present case it reflected the specific context of prior bilateral surgery for mastitis and symmetric, imaging-benign subareolar lesions, and short-interval follow-up with a low threshold for tissue diagnosis remained essential. When lesions are large, symptomatic, or ruptured—as here—surgical excision with removal of the cyst wall is appropriate and provides a definitive diagnosis [25]. Some authors caution that core-needle biopsy of an EIC may provoke rupture or inflammation, which supported the decision to proceed directly to excision in this symptomatic case. Although malignant transformation to squamous cell carcinoma is rare, it is well documented [6]. The commonly cited incidence of approximately 0.011% to 0.045% refers to cutaneous epidermal cysts in general rather than to breast lesions specifically, for which no reliable incidence is available. Several features present in our patient, including masses larger than 2 cm, heterogeneous content, and persistent bloody, purulent discharge, are among the signs that should prompt exclusion of malignancy; histologic confirmation of all excised suspected EICs is therefore prudent [6].

### 3.5. Limitations

This report has several limitations. First, as a single case, its findings cannot be generalized to all breast EICs, and the diagnostic value of the described sonographic features was not validated against pre-operative cytology or histology. Second, the initial and follow-up ultrasound examinations were performed on different systems (LOGIQ E10 and Aixplorer, respectively); differences in transducer characteristics, spatial resolution, dynamic range, and image processing may have contributed to the apparent increase in conspicuity of the onion-ring pattern at follow-up, independent of any true change. Third, shear-wave elastography was performed on the left lesion only, so an elastographic comparison between the ruptured and unruptured cysts could not be made within this patient. Fourth, the nipple discharge was not evaluated by cytology or galactography, so a concurrent ductal source could not be formally excluded. Finally, the proposed implantation mechanism remains a hypothesis, as the operative and pathology records of the prior mastitis surgery were no longer available.

## 4. Conclusions

A bilateral subareolar EIC of the breast is rare and may arise years after breast surgery, possibly through implantation of epidermal elements. Within a single patient, an unruptured cyst may retain the lamellated onion-ring sign—which can become more conspicuous on short-interval follow-up—while a ruptured cyst loses this pattern and shows inflammatory features, high elastographic stiffness, and appearances that may mimic malignancy or infection. Because these imaging findings are not specific, integrating grayscale, color Doppler, and elastographic findings together with histologic confirmation is important for accurate recognition and appropriate management. In this patient, excision was both diagnostic and curative: the nipple discharge resolved, and there was no recurrence or complication over two years of follow-up.

## Data Availability

The data presented in this study are available on request from the corresponding author. The data are not publicly available due to privacy restrictions, given the potential risk of re-identification from clinical imaging.
